# Toward sleep health as a focus of physical therapy practice: one lecture can positively impact sleep knowledge and beliefs in entry-level students

**DOI:** 10.1186/s12909-023-05008-3

**Published:** 2024-01-10

**Authors:** Catherine F. Siengsukon, Allison Glaser, Eryen Nelson

**Affiliations:** Department of Physical Therapy, Rehabilitation Science, and Athletic Training, University of Kansas Medical Center, 3901 Rainbow BlvdMail Stop 2002, Kansas City, KS 66160 USA

**Keywords:** Sleep health, Entry-level physical therapist education, DPT students, Sleep knowledge, Sleep beliefs

## Abstract

**Background:**

Challenges to integrating health promotion including sleep health into entry-level physical therapist curricula include lack of faculty expertise, time, and support. A lecture provided by a content expert may mitigate such challenges. The purpose of this study was to determine if a sleep education session impacts Doctor of Physical Therapy students’ knowledge and beliefs about sleep.

**Methods:**

Faculty shared the opportunity to participate in the study 1–3 days prior to the remotely-provided lecture including sleep health assessment and interventions. The survey included demographics, a sleep health knowledge question, 11 questions on “What I think about sleep as a professional”, and the 20-item Sleep Beliefs Scale. McNemar’s and paired sample t-tests determined change in knowledge and beliefs.

**Results:**

209 individuals (70% female, 86% Caucasian, 25.5 ± 3.4 years old) completed the pre-lecture survey, and 137 individuals completed the post-lecture survey. There was an increase in knowledge about sleep health (*p* < .001) and change in Sleep Beliefs Scales score (*p* < .001).

**Conclusions:**

A single remotely provided sleep education session increased DPT students’ knowledge and changed their beliefs about sleep. Future studies should determine if these positive beliefs about sleep translate into clinical practice and enhance patient outcomes.

## Background

Sleep has a critical function in overall health and recovery following injury. Additionally, sufficient sleep is necessary for important processes such as learning, memory consolidation, pain modulation, and cognitive functioning [1, 2]. Insufficient sleep is associated with an increased risk of several chronic health conditions such as diabetes, obesity, cardiovascular disease and depression, all of which contribute to increased mortality [3, 4]. Therefore, promoting optimal sleep impacts the health, wellbeing, and recovery of clients receiving physical therapy (PT) services and may prevent or delay the onset of chronic health conditions. Furthermore, sleep health is increasingly recognized as a key pillar of health promotion, including within the PT profession [5–8], and is a “priority health behavior” in entry-level physical therapist education programs [9]. The APTA’s House of Delegates recently formally recognized the physical therapists’ role in sleep health promotion [10].

Inadequate sleep is a common problem in the United States; approximately 1/3 of adults sleep less than the recommended 7 h, 81% of adults have taken a nap in the past three month (suggesting insufficient nighttime sleep), and about 75% of older adults experience symptoms of insomnia [11]. While there are limited studies that have examined sleep disturbances in people receiving physical therapy services [12–14], the prevalence is likely high due to the common occurrence of sleep disturbances in people with musculoskeletal injuries, neurologic disorders, pain, and other diagnoses commonly treated by physical therapists. In two studies of individuals with low back pain receiving physical therapy, 92% [11] and 93% [12] of participants reported poor sleep quality, respectively. In a sample of 88 patients receiving physical therapy services, 51% scored a 10 or higher on the Insomnia Severity Index, indicating increased risk of insomnia, and 78% scored > 5 on the Pittsburgh Sleep Quality Index, indicating poor sleep quality [14]. These high insomnia scores and prevalence of poor sleep quality in those seeking PT services emphasizes the need for physical therapists to screen for sleep disorders and to provide sleep health education to these patients.

Despite the critical role of sleep in health and recovery, physical therapists are not often assessing or addressing their clients’ sleep issues, with the most common reason being lack of knowledge on *how* [15, 16]. Lack of sleep assessment and sleep intervention resources and lack of physical therapist knowledge were barriers reported in a survey of physical therapists assessing barriers and facilitators to implementing sleep health into outpatient physical therapy practice [17]. A survey of Jordanian physical therapists’ knowledge and attitudes about sleep showed that 100% of respondents agreed that poor sleep impacts people’s health, and 95% think physical therapists should receive education about sleep [16]. However, 75% of respondents stated that they did not receive education about sleep during their PT education, and 86% did not receive sleep education following graduation from their PT program [16]. These results closely mirror the results from a similar survey study conducted in 2015 with physical therapists in the United States [15]. These survey studies indicate a need for sleep education to be further embedded into entry-level physical therapist education programs.

An opportunity to progress the physical therapy profession forward in our role in sleep health is to integrate sleep health education in entry-level physical therapist program curricula. A major limitation to integrating health promotion (not specific to sleep health) and other related topics into entry-level physical therapist curricula was the lack of a faculty content expert in the area [5, 18]. Other limitations to integrating health promotion content into PT curricula include a perceived lack of time and support [18]. With more common use of video conferencing technology to provide education sessions remotely, these limitations could perhaps be mitigated by a sleep health expert providing an education session(s) or consulting with faculty regarding integration of sleep health content into entry-level physical therapist education programs. Therefore, the purpose of this study was to determine if a sleep education session delivered by a physical therapist board certified in behavioral sleep medicine impacts Doctor of Physical Therapy students’ knowledge and beliefs about sleep.

## Methods

This study was conducted in accordance with the Declaration of Helsinki and with approval from and in accordance with the University of Kansas Medical Center’s Institutional Review Board (STUDY00148219). CS was invited by six faculty to give a remotely-provided lecture regarding sleep health in their entry-level DPT programs (4 based in Midwest and 2 based in East Coast; 1 includes hybrid model) between July 2022 and March 2023. CS emailed the faculty standardized IRB-approved information regarding opportunity for the DPT students to participate in the study including a link to a survey in REDCap (Research Electronic Data Capture) hosted at the University of Kansas Medical Center [19, 20]. The faculty then shared with their respective students the opportunity to participate in the study 1–3 days prior to the lecture. Consent was obtained electronically within REDCap by the participant selecting “Yes, I agree to participate in the study” after being provided with written consent document and signing their name. The survey consisted of 2 sections: 1. Demographics section consisting of eight questions in total including age, sex identity, ethnicity, race, age, and prior education about sleep, and 2. Knowledge and beliefs about sleep section including 1 question to rate their knowledge about sleep health from 0–100 with 0 = “no knowledge” and 100 = “expert”, and 11 questions on “What I think about sleep as a professional” rated on a 6-point Likert Scale of “Strongly Disagree”, “Disagree”, “Neither”, “Agree”, “Strongly Agree”, and “Unsure” based on Siengsukon et al. prior survey [15], and the Sleep Beliefs Scale which consists of 20 items of behaviors rated as having a “Positive Effect”, “Neither Effect” or “Negative Effect” on sleep quality and/or quantity with a total score ranging from 0–20 with a higher score indicating more positive beliefs about sleep [21]. Students who completed the pre-lecture survey were sent two days later an automated email with link to complete the post-lecture survey. The post-lecture survey consisted of the same questions on “What I think about sleep as a professional” and the Sleep Beliefs Scale as well as two additional questions to gather feedback.

The objectives and primary content for the sleep lecture were similar across programs and included why sleep is a critical component of chronic disease prevention and health promotion, selecting and implementing appropriate screening tools for the most common sleep disorders, and strategies into practice to promote clients’ sleep health. The lecture was provided remotely via Zoom or Teams and ranged in length from 90–180 min depending on the time allotment for each course, with the primary difference in length being length of time for question and answer and case discussion.

Descriptive statistical analyses were conducted; mean and standard deviations were calculated for continuous variables, and frequency distributions were calculated for categorical data. McNemar’s test was used to determine change in percentage of those who agreed (responded “Strongly Agree” or “Agree”) or disagreed/neutral (responded “Neither”, “Unsure”, “Disagree”, and “Strongly Disagree”) with survey questions pre- to post-lecture in individuals who completed both surveys. Paired samples t-tests were used to assess for change in knowledge and scores on the Sleep Beliefs Scale. Alpha was set at 0.05.

## Results

A total of 365 DPT students were invited by their respective faculty member to participate in the study. The average length of lecture was 123.3 min (SD 30.1). While 212 individuals started the pre-lecture survey, 209 individuals completed the survey, and 141 individuals started the post-lecture survey, but 137 individuals completed the survey. Of the 209 individuals who completed the survey, 70% were female, 92% were not Hispanic or Latino, 86% were Caucasian, and the average age was 25.5 (SD 3.4) years old (Table 1). All included in analysis completed the pre-lecture survey prior to the start of the survey, and all completed the post-lecture survey within 1 week of the lecture. Fifty-two percent (*n* = 108) reported they had received prior education about sleep. The most common source of prior education was lectures during the students’ undergraduate experience.

**Table 1:** Demographics

	n (%)
Sex	
Male	63 (30)
Female	146 (70)
Age (mean, SD)	25.5 (3.4)
Ethnicity	
Not Hispanic or Latino	193 (92)
Hispanic	14 (7)
Unknown	2 (1)
Race^a^	
White	180 (86)
African American	7 (3)
American Indian or Alaskan Native	3 (1)
Asian	17 (8)
Native Hawaiian or other Pacific Islander	0
Multiracial	5 (2)
Other	6 (3)
Prior education about sleep^a^	
No	101 (48)
Yes	108 (52)
If yes, type of education received (n):	
Work experience	6
Lecture(s) in undergrad program	58
During PT program	22
Continuing education	4
Self-education	9
Experience as patient	6

Demographics (*n* = 209); ^a^able to select all that apply

Pre-lecture, the average rating for sleep health knowledge was 56.8 (SD 14.8; range 10–85) and average score on the Sleep Beliefs Scale was 16.0 (SD 2.4; range 8–20; Table 2). Over 90% of respondents “Agreed” or “Strongly Agreed” with 9 out of the 11 statements regarding beliefs about sleep as a professional (Table 3) prior to the lecture. Two items (“PTs should perform objective assessments (such as questionnaires) to assess their clients' sleep habits and quality” and “PTs should counsel patients regarding methods to improve sleep quality”) were answered with 73% and 79%, respectively, “Agreed” or “Strongly Agreed”. Of those who completed the post-lecture survey, over 95% “Agreed” or “Strongly Agreed” with all 11 statements regarding beliefs about sleep as a professional (Table 4). Of participants who completed both surveys, there was a significant increase in the percentage of participants who agreed with 3 of the 11 statements regarding beliefs about sleep as a profession (“PTs should perform objective assessments (such as questionnaires) to assess their clients' sleep habits and quality”, “PTs should counsel patients regarding methods to improve sleep quality”, and “Assessing my patients' sleep habits and quality is important”; Table 5). There was also a significant increase in knowledge about sleep health (*p* < 0.001) and Sleep Beliefs Scales score (*p* < 0.001).

**Table 2:** Pre- and Post-lecture knowledge about sleep health and beliefs (*n* = 137)

	Pre-lecture	Post-lecture	
	Mean (SD) Range	Mean (SD) Range	*p*-value
Knowledge about sleep health	56.8 (14.8) 10–85	71.9 (10.2) 40–92	< .001
Sleep Beliefs Scale	16.0 (2.4) 8–20	18.3 (1.8) 12–20	< .001

**Table 3:** All respondents pre-lecture beliefs about sleep as a professional

	Strongly Agree	Agree	Neither	Disagree	Strongly Disagree	Unsure
	n (%)	n (%)	n (%)	n (%)	n (%)	n (%)
Sleep is important to people's health	204 (98)	5 (2)	0	0	0	0
Poor sleep is associated with impaired function	161 (77)	46 (22)	0	1 (.5)	1 (.5)	0
I should routinely ask patients about sleep problems	97 (46)	102 (49)	7 (3)	2 (1)	0	1 (.5)
Sleep disorders may contribute to medical problems	142 (70)	63 (30)	2 (1)	0	0	2 (1)
PTs should ask their patients about their sleep habits and sleep quality	121 (58)	81 (39)	5 (2)	0	0	1 (.5)
PTs should perform objective assessments (such as questionnaires) to assess their clients' sleep habits and quality	46 (22)	106 (51)	41 (20)	6 (3)	0	8 (4)
PTs should counsel patients regarding methods to improve sleep quality	68 (33)	97 (46)	29 (14)	6 (3)	0	9 (4)
PTs should counsel patients on positioning to improve sleep	113 (54)	79 (38)	11 (5)	1 (.5)	0	5 (2)
Assessing my patients' sleep habits and quality is important	92 (44)	102 (49)	10 (5)	1 (.5)	0	4 (2)
Addressing sleep issues may impact physical therapy outcomes	121 (58)	84 (40)	2 (1)	0	0	1 (.5)
I think DPT students should receive education about sleep	138 (66)	66 (32)	3 (1)	0	0	2 (1)

Before lecture responses (*n* = 209) regarding “What I think about sleep as a professional”

**Table 4:** Respondents post-lecture beliefs about sleep as a professional

	Strongly Agree	Agree	Neither	Disagree	Strongly Disagree	Unsure
	n (%)	n (%)	n (%)	n (%)	n (%)	n (%)
Sleep is important to people's health	134 (98)	2 (1)	0	0	1 (1)	0
Poor sleep is associated with impaired function	128 (93)	8 (6)	0	0	1 (1)	0
I should routinely ask patients about sleep problems	116 (85)	20 (15)	0	0	1 (1)	0
Sleep disorders may contribute to medical problems	122 (89)	14 (10)	0	0	1 (1)	0
PTs should ask their patients about their sleep habits and sleep quality	113 (82)	23 (17)	0	0	1 (1)	0
PTs should perform objective assessments (such as questionnaires) to assess their clients' sleep habits and quality	101 (74)	30 (22)	4 (3)	1 (1)	1 (1)	0
PTs should counsel patients regarding methods to improve sleep quality	95 (69)	40 (29)	1 (1)	0	1 (1)	0
PTs should counsel patients on positioning to improve sleep	111 (82)	21 (15)	3 (2)	0	1 (1)	0
Assessing my patients' sleep habits and quality is important	110 (80)	25 (18)	1 (1)	0	1 (1)	0
Addressing sleep issues may impact physical therapy outcomes	115 (85)	20 (15)	0	0	1 (1)	0
I think DPT students should receive education about sleep	113 (82)	21 (15)	2 (1)	0	1 (1)	0

After lecture responses (*n* = 137) regarding “What I think about sleep as a professional”

**Table 5:** Pre- and post-lecture beliefs about sleep as a professional

	Pre-lecture		Post-lecture		
	Agree	Disagree/ Neutral	Agree	Disagree/ Neutral	
	n (%)	n (%)	n (%)	n (%)	*p*-value
Sleep is important to people's health	137	0	136	1	1.00
Poor sleep is associated with impaired function	135	2	136	1	1.00
I should routinely ask patients about sleep problems	132	5	136	1	1.00
Sleep disorders may contribute to medical problems	133	4	136	1	1.00
PTs should ask their patients about their sleep habits and sleep quality	133	3	136	1	1.00
PTs should perform objective assessments (such as questionnaires) to assess their clients' sleep habits and quality	101	36	131	6	< .001
PTs should counsel patients regarding methods to improve sleep quality	107	30	135	2	< .001
PTs should counsel patients on positioning to improve sleep	124	13	132	4	1.00
Assessing my patients' sleep habits and quality is important	125	12	135	2	.006
Addressing sleep issues may impact physical therapy outcomes	133	3	135	1	.607
I think DPT students should receive education about sleep	133	4	134	3	1.00

Responses “Strongly Agree” and “Agree” combined into “Agree”; Reponses “Neither”, “Unsure”, “Disagree”, and “Strongly Disagree” combined into “Disagree/Neutral”. N = 137 who completed both the pre-lecture and post-lecture survey although n may be less for certain items if respondent left blank

## Discussion

This is the first study to assess DPT students’ knowledge and beliefs about sleep and demonstrated that a single remotely provided sleep education session increased their self-reported knowledge and changed their beliefs about sleep. The large majority of participants had positive beliefs regarding sleep prior to the sleep lecture, and results indicated opportunities to grow knowledge and skills regarding sleep assessment and methods to improve sleep health. Future studies should determine if these positive beliefs about sleep translate into clinical practice and ultimately to enhanced patient outcomes.

It was surprising that > 90% of respondents agreed with 9 of the 11 statements pertaining to perceptions about sleep as a professional, including “I should routinely ask patients about sleep problems” and “PTs should ask their patients about their sleep habits and sleep quality”. A smaller percentage (albeit still relatively large at 73% and 79%) agreed with the statements “PTs should perform objective assessments (such as questionnaires) to assess their clients' sleep habits and quality” and “PTs should counsel patients regarding methods to improve sleep quality”. This large percentage of positive beliefs about sleep before the sleep health lecture may be due to the majority of participants having had prior education about sleep, the integration and addition of prevention and health promotion (which includes sleep health) into entry-level physical therapist education programs in recent years [9, 18, 22] as well as the passage of the House of Delegates position on the role of physical therapists in sleep health [10].

It is possible that the 73% and 79% who agreed regarding objective assessments and counseling patients is due to lack of knowledge on which specific objective assessments to utilize, lack of knowledge on specific education to provide to improve sleep quality, and lack of skills to coach patients to change their sleep behavior. Fortunately, one lecture on sleep health which included content on assessment and sleep health promotion techniques (but did not include content on coaching skills or behavior change techniques) was sufficient to change their beliefs regarding these two items, enhance their score on the Sleep Beliefs Scale, and increase their perceived sleep health knowledge. It will be important to determine in the future if these positive beliefs about sleep translate into assessing sleep issues and providing sleep health promotion techniques in clinical practice. Another opportunity for future research is to increase scalability of the sleep lecture by determining if a recorded lecture would produce similar positive results.

To our knowledge, this is the first study to assess if a sleep lecture can change DPT students’ knowledge and beliefs about sleep. However, three studies have examined if a single pain neuroscience education lecture (ranging from 45–120 min) changes in knowledge, attitudes, and beliefs about pain in physical therapy students [23–25]. All three demonstrated an improvement in physical therapy students’ knowledge regarding pain from before to immediately and at 6 months following the lecture, and two demonstrated a change in the students attitudes and beliefs regarding chronic pain [24, 25]. These studies are in line with the results of our study and illustrate that a single lecture is sufficient to enhance knowledge, beliefs, and attitudes about the topic.

A main limitation of this study is that participants all attend entry-level physical therapist education programs that invited a physical therapist who is an expert in behavioral sleep medicine to provide a lecture to their students as part of a class. It is likely that the faculty who invited the lecturer and the programs they are employed by recognize the importance of sleep for health and wellbeing or they would not have invited the lecturer, although we did not examine the beliefs and perceptions about sleep of the faculty inviter. This may bias the sample and limit generalizability. Also, the programs were mostly based in the Midwest which also may impact results and may limit generalizability. In addition, there was a large attrition between the number of individuals who completed the pre-lecture survey and those that completed the post-lecture survey. We suspect this is because the post-lecture survey was sent by the REDCap system so the email may have looked suspicious or thought to be spam and thus ignored. Other limitations are measuring self-perception of knowledge rather than actual sleep knowledge, not measuring retention of knowledge, inability to measure actual change in practice upon entry into the workforce, and not collecting information on year in the DPT program. Future studies should address these limitations and expand our current body of knowledge. In addition, a future study should consider determining if a lecture on sleep health can change knowledge and beliefs in practicing physical therapists. Also, a future study should consider using a recorded lecture to standardize the content and potentially to increase access to the content.

## Conclusions

Our preliminary findings support that even a single lecture on sleep health to a sample of Doctor of Physical Therapy students can increase their knowledge and positive beliefs about the role of sleep on health. In addition to replicating our findings, future studies are needed to establish whether providing tools for maximizing sleep health in addition to strengthening sleep knowledge and beliefs, can increase client health and physical therapy outcomes.

## Data Availability

The datasets used and/or analyzed during the current study available from the corresponding author on reasonable request.
